# Cardiovascular magnetic resonance of pulmonary artery growth and ventricular function after Norwood procedure with Sano modification

**DOI:** 10.1186/1532-429X-10-34

**Published:** 2008-07-06

**Authors:** D Scott Lim, Benjamin B Peeler, G Paul Matherne, Christopher M Kramer

**Affiliations:** 1 Department of Pediatrics, University of Virginia, Charlottesville, USA; 2 Department of Surgery, University of Virginia, Charlottesville, USA; 3 Departments of Medicine & Radiology, University of Virginia, Charlottesville, USA

## Abstract

For hypoplastic left heart syndrome (HLHS), there have been concerns regarding pulmonary artery growth and ventricular dysfunction after first stage surgery consisting of the Norwood procedure modified with a right ventricle-to-pulmonary artery conduit. We report our experience using cardiovascular magnetic resonance (CMR) to determine and follow pulmonary arterial growth and ventricular function in this cohort.

Following first stage palliation, serial CMR was performed at 1 and 10 weeks post-operatively, followed by cardiac catheterization at 4 – 6 months. Thirty-four of 47 consecutive patients with HLHS (or its variations) underwent first stage palliation. Serial CMR was performed in 20 patients. Between studies, ejection fraction decreased (58 ± 9% vs. 50 ± 5%, p < 0.05). Pulmonary artery growth occurred on the left (6 ± 1 mm vs. 4 ± 1 mm at baseline, p < 0.05) but not significantly in the right. This trend continued to cardiac catheterization 4–6 months post surgery, with the left pulmonary artery of greater size than the right (8.8 ± 2.2 mm vs. 6.7 +/- 1.9 mm, p < 0.05). By CMR, 5 had pulmonary artery stenoses initially, and at 2 months, 9 had stenoses. Three of the 9 underwent percutaneous intervention prior to the second stage procedure.

In this cohort, reasonable growth of pulmonary arteries occurred following first stage palliation with this modification, although that growth was preferential to the left. Serial studies demonstrate worsening of ventricular function for the cohort. CMR was instrumental for detecting pulmonary artery stenosis and right ventricular dysfunction.

## Background

Hypoplastic left heart syndrome is a congenital cardiac malformation involving hypoplasia of the ascending aorta, aortic valve atresia or stenosis, a hypoplastic left ventricle, and mitral atresia or hypoplasia[1]. Until the past few years, the standard surgical approach to hypoplastic left heart syndrome has either been a staged surgical palliation, with the first step being the modified Norwood procedure[2], or cardiac transplantation[3,4]. The first stage surgical palliation involves aortic arch augmentation, atrial septectomy, and an aorto-pulmonary shunt for pulmonary blood flow. While surgical survival has improved at many institutions, the overall surgical mortality of this first stage of palliation remains significant[5,6]. Recently, a large number of institutions (including the University of Virginia) have switched to the "Sano" modification of the Norwood procedure, with improved short-term results in some, but not all centers[7,8]. This "Sano" modification of the Norwood procedure uses a valveless right ventricle-to-pulmonary artery conduit in place of the aorto-pulmonary shunt for pulmonary blood flow. However, concern remains regarding the growth of the pulmonary arteries and right ventricular function with this surgical modification, which involves a ventriculotomy in the systemic right ventricle[9]. While echocardiography has become the primary imaging modality in pediatric cardiology, it is limited in its ability to image the branch pulmonary arteries after this surgical intervention.

At the University of Virginia, because of these concerns it became our clinical practice to perform a cardiovascular magnetic resonance (CMR) study prior to discharge and at two months of age, followed by cardiac catheterization at 4 – 6 months of age, in children with hypoplastic left heart syndrome. The next stage of surgical palliation was usually performed after 4 – 6 months of age, and involved ligation and division of the right ventricle-to-pulmonary artery conduit, and creation of a superior vena cava-to-pulmonary artery anastomosis. If CMR defined any significant pulmonary artery stenosis or coarctation, earlier transcatheter intervention was undertaken.

The intent of this study was to report on the CMR assessment of growth of the pulmonary arteries and right ventricular function after the Norwood procedure with Sano modification for hypoplastic left heart syndrome.

## Methods

**Figure 1:**
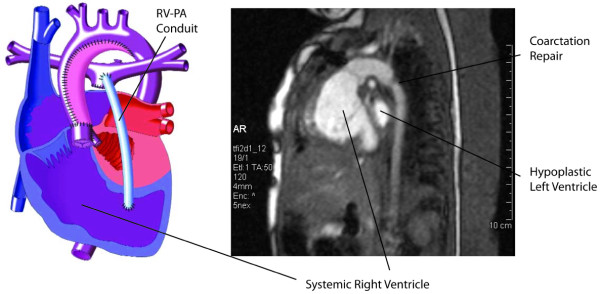
**Left-sided drawing demonstrates surgical anatomy after Norwood procedure with Sano modification, in which pulmonary blood flow is supplied by right ventricle-pulmonary artery conduit as labeled.** Right-sided CMR image shows site of coarctation repair and systemic right ventricle. (Drawing reprinted with permission from Scientific Software Solutions, Inc.)

Approval for the study was obtained from the University of Virginia Institutional Review Board Human Investigations Committee. We retrospectively reviewed the results of all children admitted following birth to the University of Virginia with the diagnosis of hypoplastic left heart syndrome (or its variations) from 2002 to the end of 2006. The time period was chosen because in mid-2002 our institution switched from the "standard" modified Norwood procedure[2,10] to the Sano modification[8,11]. (Figure 1) The procedure was carried out with arterial cannulation via the innominate artery and regional perfusion with minimal use of deep hyperthermic circulatory arrest. The neoaortic arch was constructed utilizing pulmonary homograft. The right ventricular to pulmonary artery conduit was constructed using either 5 or 6 mm ringed polytetrafluoroethylene graft, running to the patient's right of the neoaorta, and a chimney patch created and anastomosed to the confluence of pulmonary arteries distally. The chimney patch was constructed using a 0.4 mm thin polytetrafluoroethylene cardiovascular patch and the ringed polytetrafluoroethylene conduit. The polytetrafluoroethylene patch was cut into an elliptical shape, and a 6 mm hole was created in the center of the patch using an aortic punch. The conduit was anastamosed to the opening in the patch using a 6-0 prolene suture, and the chimney patch was then anastamosed to the pulmonary artery confluence. A 4.8 mm aortic punch was used to create a ventriculotomy in the right ventricular outflow tract. This right ventriculotomy is used for the proximal anastomotic site for the right ventricle-to-pulmonary artery conduit. The conduit size was 5 mm in patients < 3.5 kg, and 6 mm in the remainder. The same surgical and cardiology team was involved in all cases.

Following an episode of unrecognized conduit and pulmonary artery stenosis leading to cardiovascular collapse, we began a clinical program of surveillance screening by CMR for the development of significant pulmonary artery stenosis or coarctation of the aorta. CMR was performed in patients prior to discharge after first stage surgery, and again at 2 months post-operatively. By prospective protocol, CMR was also performed at 1 year of age in patients with persistent late ventricular dysfunction as defined by follow-up echocardiography.

Studies were performed with general anesthesia and endotracheal intubation, which allowed the patients to remain still for the studies. Cardiac CMR was performed on a 1.5 Tesla Sonata scanner (Siemens Medical Solutions, Erlangen, Germany). The patient was placed supine in a two-channel head coil with monitoring of electrocardiogram, blood pressure, and oxygen saturation. Steady state free precession (SSFP) cine imaging was performed with repetition time of 2.7 ms, echo time 1.3 ms, temporal resolution 19 ms, slice thickness 4 mm, flip angle 27°, field of view 200 mm, matrix 76 × 192, and 4–5 signal averages during free breathing. Spatial resolution for SSFP was 2.6 × 1.0 × 4.0 mm. Axial and right ventricular short-axis image stacks were obtained without a gap. In addition, velocity encoded gradient echo cine imaging was obtained perpendicular to the proximal portion of the right ventricle-to-pulmonary artery conduit and in the plane of the proximal branch pulmonary arteries with repetition time of 4 ms, echo time of 3.6 ms, temporal resolution of 29 ms, slice thickness 4 mm, flip angle 30°, field of view 200 mm, matrix 93 × 256, velocity encoded at 300 cm/s, and 3 signal averages during free breathing. Spatial resolution for velocity encoded images was 2.2 × 0.8 × 4.0 mm. Finally, a 3-dimensional contrast-enhanced CMR angiogram was obtained during infusion of 0.2 mM/kg of gadolinium-DTPA. The angiogram was obtained in 1 slab with repetition time of 3.75, echo time 1.4 ms, slice thickness of 0.9 mm, flip angle 30°, field of view 200 mm, matrix 75 × 320, and 6 signal averages. Spatial resolution for CMR angiogram images was 2.7 × 0.6 × 0.9 mm.

Dedicated analysis software (Argus, Siemens Medical Solutions, Malvern, PA) was utilized to calculate end-diastolic ventricular volumes and systolic function from the short axis SSFP cine images. The anterior-posterior diameter of the branch pulmonary arteries was measured from the velocity encoded cine images and confirmed by the CMR angiogram, and the point of measurement was at the hilum. SSFP cine images were not used for this measurement because of off-resonance flow effects that prevented adequate visualization of the branch pulmonary arteries. Significant pulmonary artery stenosis or coarctation were defined by CMR as an abrupt luminal narrowing > 40%[12]. Right ventricle-to-pulmonary artery conduit regurgitation volume and fraction was measured from the velocity encoded imaging obtained perpendicular to the conduit using Argus software. The CMR angiogram was viewed on the Argus 3-dimensional workstation as a multiplanar reformat and maximum intensity projection.

**Figure 2:**
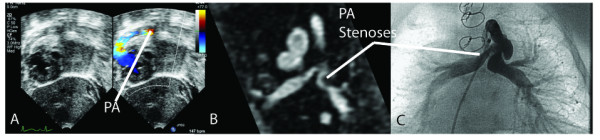
**Panel A illustrates the difficulty in imaging the branch pulmonary arteries by transthoracic echocardiography. **Turbulence from the normally increased flow across the right ventricle-pulmonary artery conduit obscures the branch pulmonary artery anatomy. Panel B is in the same patient and branch pulmonary artery stenoses are demonstrated clearly by CMR, which is confirmed at time of cardiac catheterization and angiography in panel C.

At time of CMR, all patients also had transthoracic echocardiography to compare estimates of ventricular function, and diagnoses of pulmonary artery stenoses and aortic coarctation. (Figure 2) Pulmonary artery stenosis was diagnosed by demonstrating a qualitative luminal narrowing on the color Doppler signal, rather than by Doppler-derived gradients as there were high velocities from the proximal conduit. Two-dimensional imaging by echocardiography was not able to evaluate the branch pulmonary artery anatomy after surgical palliation with the right ventricle-pulmonary artery conduit. Echocardiographic definition of coarctation was a peak-instantaneous Doppler-derived gradient in the aorta of > 20 mmHg using both proximal and distal velocities in the complete modified Bernoulli equation [13].

All patients underwent cardiac catheterization prior to the second stage surgery, which was intended at 4–6 months of age. Right ventricular ejection fraction was calculated by calibrated biplane Simpson's rule [14]. Given the difficulty with interpretation of multiple pressure gradients in series, and that a large gradient is normally present across the right ventricle-pulmonary artery conduit, only the presence of a gradient at the site of angiographic luminal narrowing in the branch pulmonary artery was used to define a significant pulmonary artery stenosis. A significant coarctation was defined by catheterization as a gradient of > 20 mmHg in the presence of angiographic narrowing[15]. Significant coarctations underwent percutaneous balloon angioplasty[16,17], and success was defined as a gradient of < 10 mmHg without significant drop in cardiac output.

The authors had full access to the data and take responsibility for its integrity. All authors have read and agree to the manuscript as written.

### Statistical Analysis

Serial measurements for patients were compared using analysis of variance for repeated measures, and comparison between patients was analyzed using unpaired *t *tests, with statistical significance defined as p < 0.05.

## Results

### Demographics

From 2002 to 2006, 47 newborns with hypoplastic left heart syndrome (or its variations, see Table 1) were admitted to the University of Virginia. Six patients received palliative care only and expired shortly thereafter. Seven patients with HLHS and high-risk features underwent hybrid palliation, and have been previously reported on by our group[18]. The remaining 34 patients underwent the Norwood procedure with Sano modification at an age of 9 +/- 8 days (range 1 – 47 days), with survival to hospital discharge in 26 of 34. Initially, the first 9 infants underwent surgical intervention, and the 6 survivors were followed without CMR screening. Following these first 9 patients, we then began the CMR screening program for the reasons noted above. For the remainder of the study period 25 infants underwent surgical palliation, of which 5 patients expired prior to hospital discharge, with cardiac CMR being performed in the remaining 20 patients. There were no complications from the anesthesia for the CMR study.

**Table 1:** Anatomic diagnoses of the cohort are listed.

**Anatomic Diagnoses**	**n**
Hypoplastic left heart syndrome	30
Heterotaxy/Unbalanced AV canal with left ventricular hypoplasia	3
Double outlet right ventricle with left ventricular hypoplasia	1

### CMR evaluation of the right ventricle

**Figure 3:**
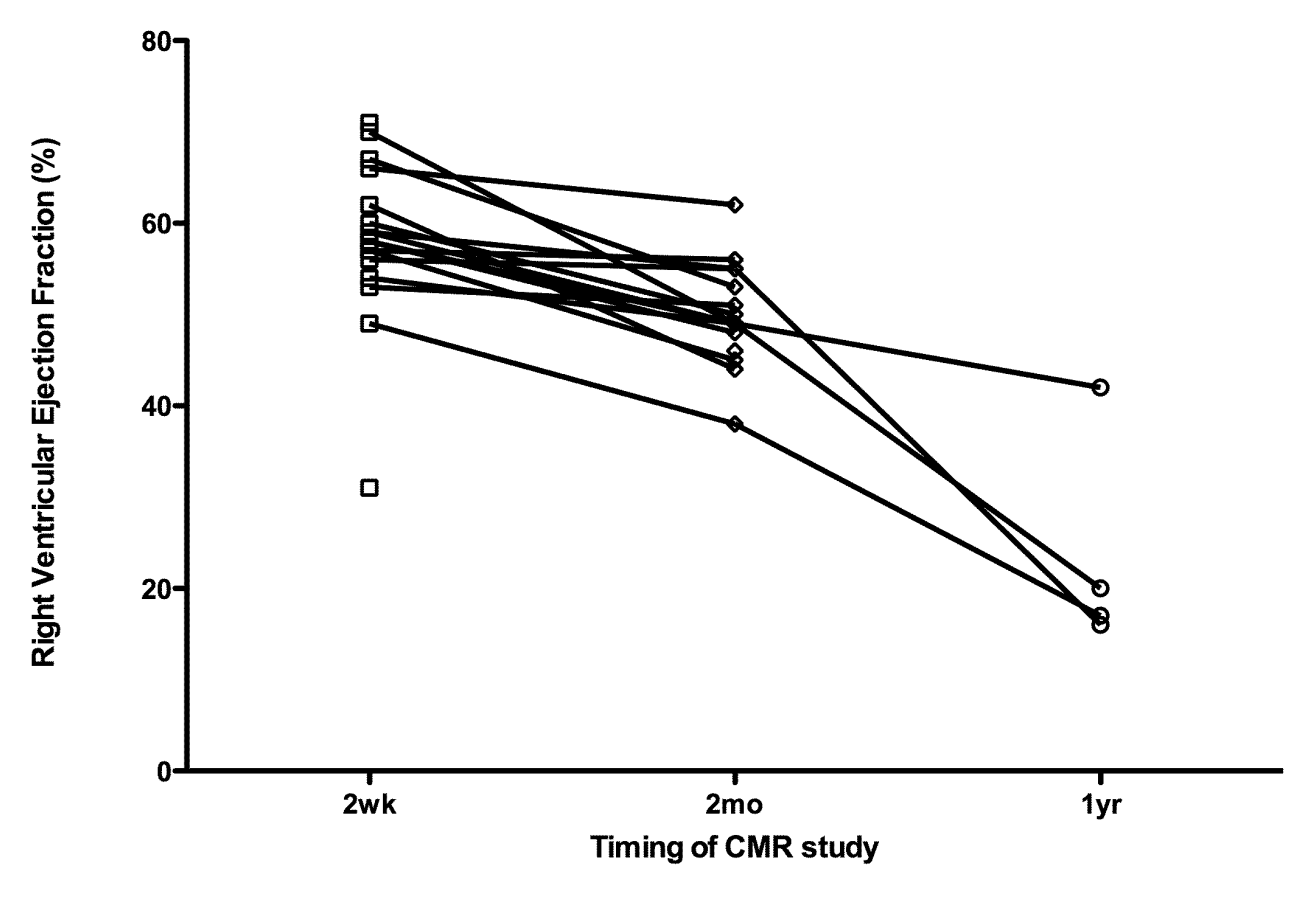
Serial measurement of right ventricular ejection fraction is shown, as assessed by cardiac CMR.

**Figure 4:**
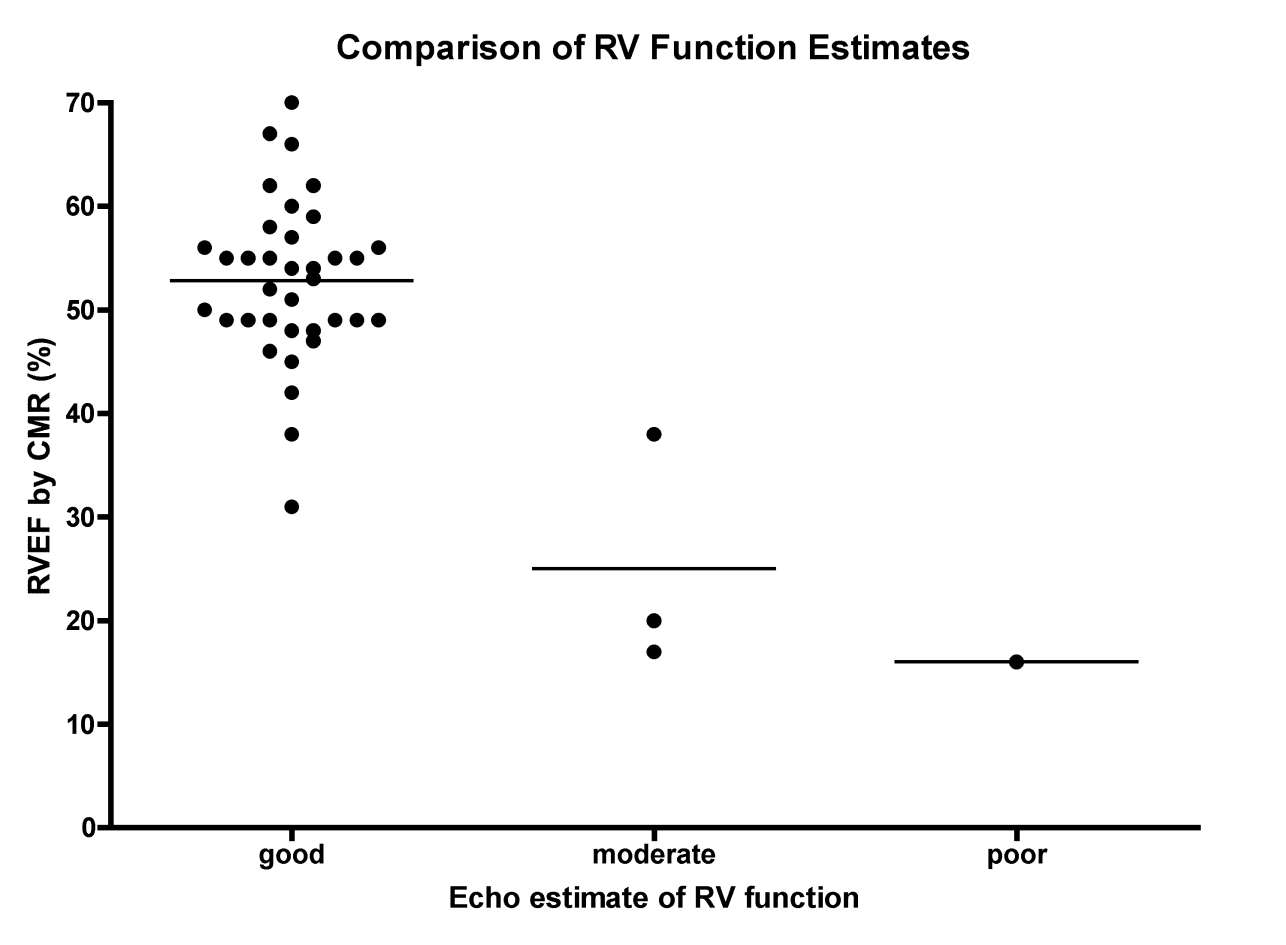
Comparison between echocardiographic and cardiac magnetic resonance imaging assessments of right ventricular systolic function is shown.

From the initial pre-discharge CMR study to the 2-month study, right ventricular volumes increased (19 ± 6 mL vs. 32 ± 5 mL, p < 0.05) but were not different compared with body growth (102 ± 33 mL/m^2 ^vs. 105 ± 33 mL/m^2^, p = NS), and right ventricular ejection fraction decreased (58.0 ± 8.9% vs. 49.9 ± 5.4%, p < 0.05). (Figure 3) The right ventricular ejection fraction as seen on the cardiac catheterization prior to the 2^nd ^stage surgical palliation was 45 ± 9%. A comparison between echocardiographic and CMR estimates of right ventricular function is shown in Figure 4. There is overlap in CMR-determined right ventricular ejection fraction between the different echocardiographic classifications of ventricular function. The CMR-determined right ventricular ejection fraction in the group with "good" systolic function by echocardiography was 53 ± 8%, with 25 ± 11% in the moderate group. The single patient with an echocardiographic estimate of "poor" function had an ejection fraction of 16%.

### CMR evaluation of the branch pulmonary arteries

**Figure 5:**
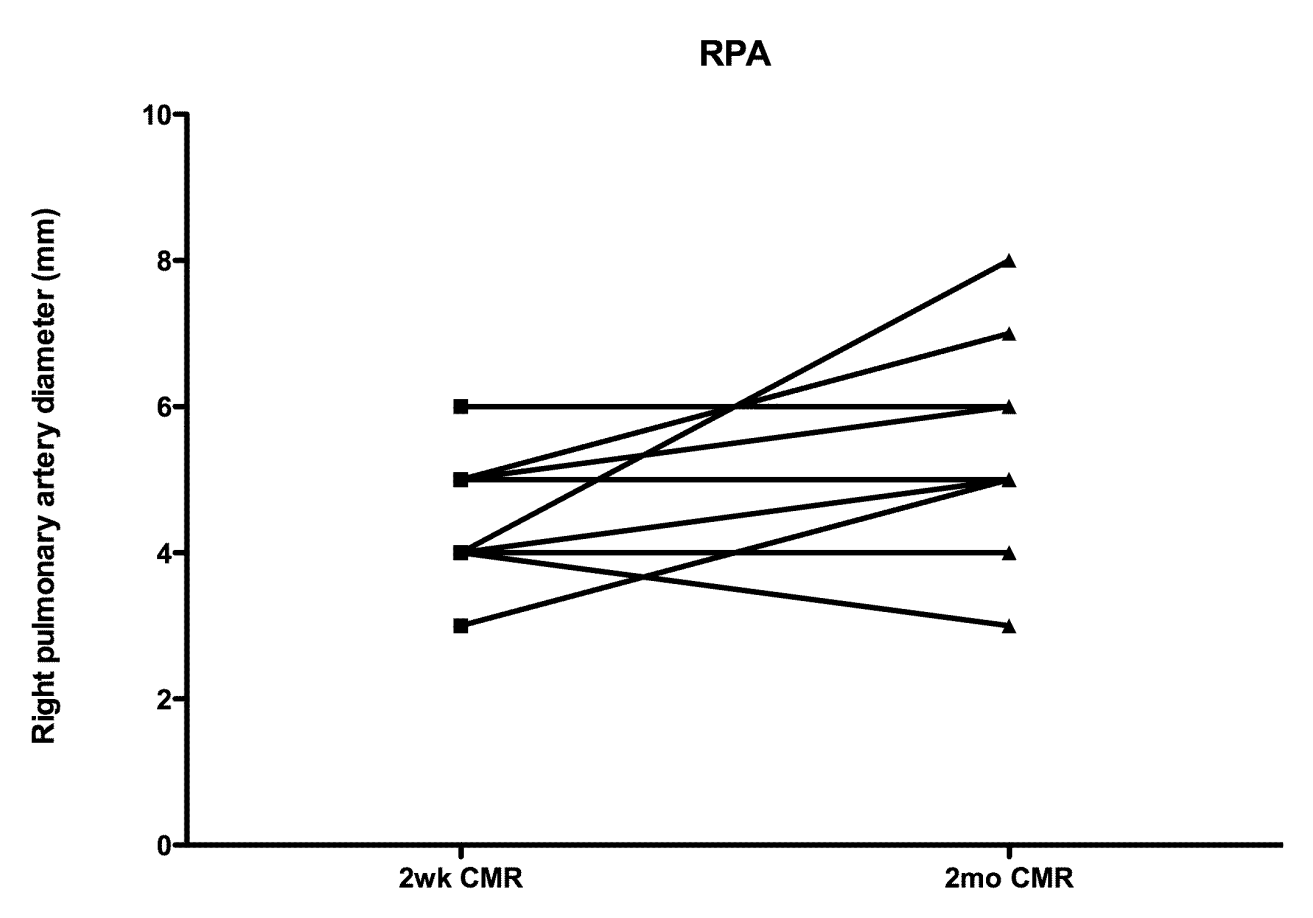
Growth in the right pulmonary artery is shown.

**Figure 6:**
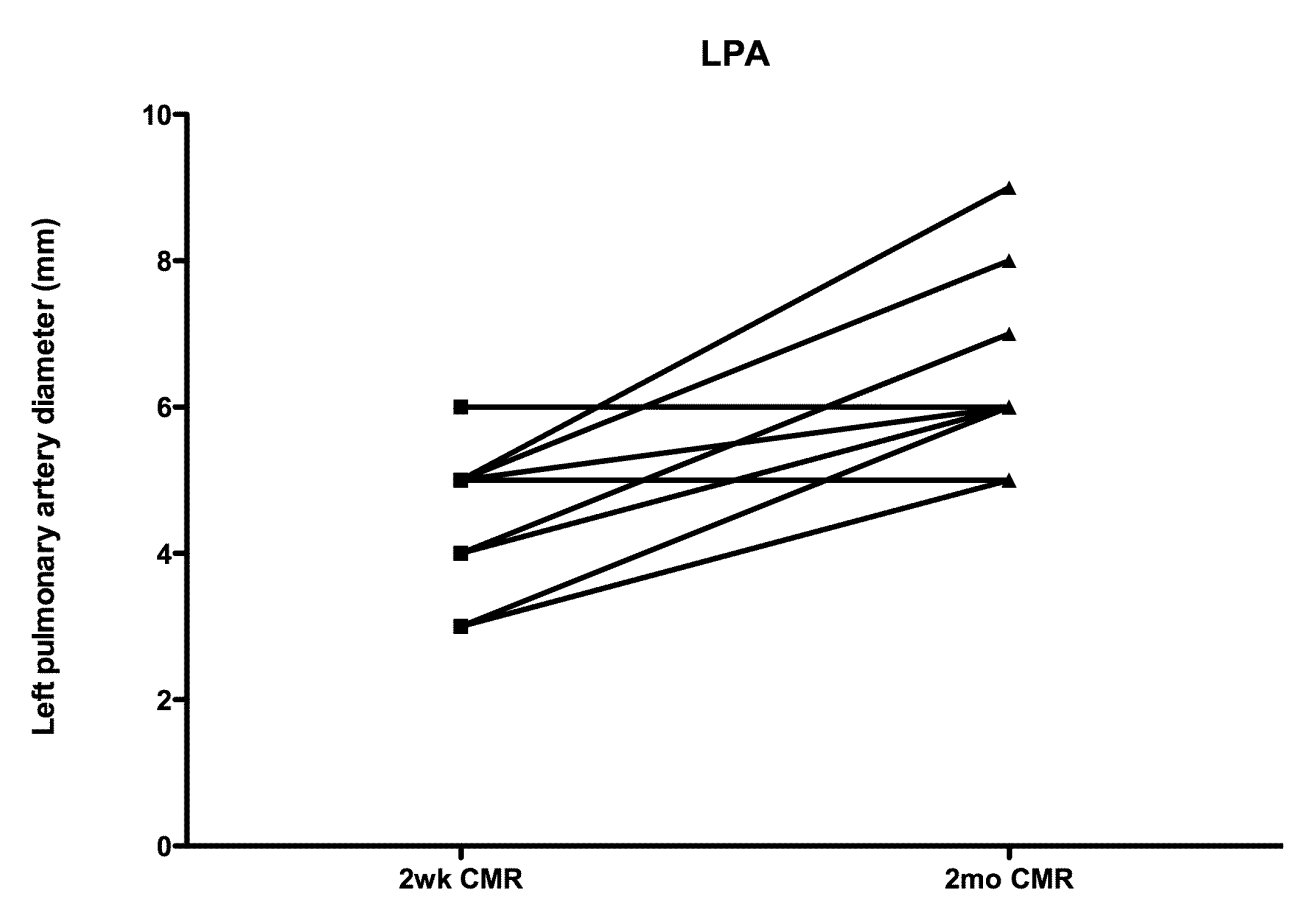
**Growth of the left pulmonary artery is shown.** There was significant growth between the first and second CMR studies.

At time of initial CMR, the right and left pulmonary arteries were of similar size (4.6 ± 0.9 mm vs. 4.4 ± 1.0 mm, p = NS). However, pulmonary artery growth occurred mainly in the left pulmonary artery (6.3 ± 1.1 mm vs.4.4 ± 1.0 mm at baseline, p < 0.05), but not significantly in the right (5.2 ± 1.5 mm vs. 4.6 ± 0.9 mm at baseline, p = NS), with the left pulmonary artery larger at time of the 2^nd ^CMR (6.3 ± 1.1 mm vs. 5.2 ± 1.5 mm for the right pulmonary artery, p < 0.05). (Figures 5 &6) This trend appeared to continue to the cardiac catheterization at 4 +/- 1 months post surgery, with the left pulmonary artery of significantly greater size than the right (8.8 ± 2.8 mm vs 6.7 ± 1.9 mm for the right pulmonary artery, p < 0.05).

By CMR, 5 patients had pulmonary artery stenoses defined at the two-week study, and at the two-month study 9/20 had pulmonary artery stenoses. Three of these underwent early percutaneous intervention for severe unilateral stenoses. On all 9 patients with CMR-defined pulmonary artery stenosis, cardiac catheterization confirmed the diagnosis. In those patients without CMR-defined pulmonary artery stenosis, no significant pulmonary artery stenosis was found at time of pre-operative cardiac catheterization.

However, the ability of echocardiography to diagnose CMR-diagnosed pulmonary artery stenosis was poor, with only a 6% sensitivity (1 of 18 stenoses were diagnosed by echocardiography). There were no false positive diagnoses of pulmonary artery stenosis by echocardiography.

There was no significant change in conduit regurgitant fraction (15 ± 4% vs. 16 ± 5%) between studies.

### CMR evaluation of the aortic coarctation

While 9 patients were defined by CMR at the 2-week study as having a coarctation, clinically it was determined that these did not require reintervention at that time. Among these 9 patients, coarctations in 6 persisted or worsened by the 2-month study, and resolved in 3. 2 additional patients developed a significant coarctation that was defined on the 2-month study. Ten of the 26 1^st ^stage survivors underwent percutaneous coarctation intervention, which was judged successful in 7, and one was felt not to have clinical coarctation despite the CMR findings. This was related to an artifact caused by a metal surgical clip. Of the patients not diagnosed by CMR with a coarctation, one was found to have a coarctation at time of the pre-operative cardiac catheterization.

Echocardiography agreed with CMR-diagnosed coarctation in 7 of 10 patients who underwent cardiac catheterization. A single patient erroneously diagnosed with a coarctation by CMR was thought not to have a significant gradient by Doppler, and two unsedated patients did not have adequate arch views to determine the presence of coarctation by echocardiography.

Those patients not having a diagnosis of recurrent coarctation had a right ventricular ejection fraction by CMR of 48 +/- 8% as compared to those with recurrent coarctation their ejection fraction was 41 +/- 12% (p = NS).

## Discussion

In this cohort of patients, CMR demonstrates an important role in the detection of surgical issues complicating the Norwood procedure with Sano modification. It was able to define pulmonary artery stenosis with 100% sensitivity and specificity, and has already been proven to be instrumental in the evaluation of right ventricular function and aortic coarctation.

Pulmonary artery growth in this cohort of infants with hypoplastic left heart syndrome after the Norwood procedure with Sano modification was asymmetric, with greater growth in the left pulmonary than the right. This asymmetry of growth may be related to both the direction of the flow from the right ventricle-to-pulmonary artery conduit, which is preferential to the left pulmonary artery, and that the right pulmonary artery must then pass under the reconstructed aortic arch. This is in contrast to the standard Norwood palliation, which involves a modified Blalock Taussig shunt, which frequently comes off of the right-sided innominate artery and inserts onto the right pulmonary artery. Previous work has shown asymmetric growth of the pulmonary arteries after the right-sided modified Blalock Taussig shunt, with the right pulmonary artery larger than the left [19]. In either type of surgical shunt, pulmonary artery asymmetry of growth may have important late implications which have not been yet defined.

For diagnosing recurrent or persistent coarctation of the aorta, the CMR has been described as highly sensitive and specific [12,20]. However, in 1 patient, the CMR incorrectly gave a suspicion of a coarctation, and in that patient the clinical diagnosis was confounded by femoral arterial occlusion. The patient had been referred for early cardiac catheterization, which determined no gradient across the aortic arch which was widely patent on angiography. Retrospective review found a surgical clip casting an artifact obscuring the aortic isthmus. It is possible that the use of a phase contrast velocity encoding sequence through the area of concern for coarctation may be useful in clarifying this. Therefore, we believe that in the absence of such imaging artifacts, CMR has an excellent ability to define the pulmonary artery and aortic anatomy after the Norwood procedure with Sano modification. However, in our patient series, there was one patient incorrectly diagnosed with coarctation by CMR. In that patient, an aortic luminal narrowing was seen on CMR. Since cuff blood pressures and Doppler echocardiography demonstrated no gradient across the coarctation site, this was felt to be hemodynamically insignificant. This is likely related to the observation that not all anatomic narrowings will produce significant hemodynamic obstructions, which may be ameliorated by vessel wall compliance and presence of collateral vessels.

In this cohort of patients, in which the single ventricle is of right ventricular morphology and in which a ventriculotomy was performed, there was a significant depression of systolic function, as measured by serial study. In addition, out of 26 initial hospital survivors, 2 subsequently underwent cardiac transplantation with another currently being listed. The indication in all 3 such patients was symptomatic heart failure. It is possible that the ventriculotomy plays a role in the development of late ventricular dysfunction, as has been noted in other centers [21]. It is also possible that recurrence of coarctation, with its attendant increased afterload on the right ventricle, may contribute to ventricular dysfunction. In our cohort of patients, we found a non-significant trend of increased ventricular dysfunction in the group with recurrent coarctation. Importantly, we took an approach of early percutaneous intervention to any new diagnosis of recurrent coarctation, on the assumption that the afterload reduction was important in these patients' management.

Both the survival to discharge and the interstage mortality in this cohort of patients following the Norwood procedure with Sano modification were similar to other reports in the current era[5,8,10,22,23], and has been associated with improved peri-operative hemodynamic stability[9]. However, it may be that this is a trade-off for later ventricular dysfunction.

Limitations of the present study include the different imaging modalities to evaluate right ventricular, pulmonary artery, and aortic anatomy and function. However, previous work has demonstrated excellent correlation between CMR and cardiac catheterization for right ventricular volumes and function[24,25], for pulmonary artery anatomy[26], and for coarctation evaluation[27].

## Conclusion

Development of significant pulmonary artery stenosis, coarctation, and right ventricular dysfunction occurs in a portion of patients undergoing the Norwood palliation with Sano modification for hypoplastic left heart syndrome. Cardiovascular magnetic resonance is instrumental in surveillance for such complications.

## Abbreviations

HLHS: Hypoplastic left heart syndrome; CMR: Cardiac magnetic resonance imaging.

## Competing interests

DSL, BBP, and GPM have no conflicts of interest to disclose. CMK has research equipment support from Siemens Medical.

## Authors' contributions

DSL designed the study, collected data, and drafted the manuscript. BBP performed the surgeries and drafted a portion of the manuscript. GPM participated in study design and data collection. CMK participated in study design and data collection, and drafted a portion of the manuscript. All authors read and approved the final manuscript.
